# Supplementary material for: Are carbon emissions trading and green financial instruments synergistic? -Comprehensive quantitative research based on content analysis
Source: PLoS One. 2024 Mar 7;19(3):e0298601. doi: 10.1371/journal.pone.0298601 (PMC10919601; doi:10.1371/journal.pone.0298601)
Supplement: S1 Data — (ZIP) [file pone.0298601.s001.zip › Data/地级市Z_F工作报告.zip!/╣π╬≈/│τ╫≤╩╨/└┤╫╘╣1⁄2╓┌║┼í╛┬φ┐╦╩2╛▌═°í┐_2018.docx]

各位代表：

现在，我代表市人民政府，向大会报告政府工作，请予审议，并请市政协委员和其他列席会议的同志提出意见。

一、 2017年工作回顾

2017年是实施“十三五”规划的重要一年，也是我市做好“两篇大文章”、打好“四大攻坚战”的第二个攻坚年。一年来，面对国内外错综复杂的发展环境，我们在自治区党委、政府和市委的坚强领导下，以迎接学习宣传贯彻党的十九大为主线，以习近平新时代中国特色社会主义思想为指导，贯彻落实新发展理念，坚持稳中求进工作总基调，全力做好“两篇大文章”、打好“四大攻坚战”，统筹推进稳增长、促改革、调结构、惠民生、防风险各项工作，努力化解经济下行压力，全市经济社会保持持续稳定发展态势，向与全国全区同步全面建成小康社会目标迈出了坚实一步。

一年来，我们主要做了以下工作：

（一）坚持稳中求进，经济基本面保持稳定。全面贯彻落实自治区稳增长政策措施，制定出台崇左市促进实体经济稳增长若干措施，助推企业降本增效，形成稳增长的强大合力。初步预计，全年地区生产总值887亿元，同比增长8.5%；财政收入55.25亿元，完成年度目标任务；规模以上工业总产值922亿元，增长23.5%；规模以上工业增加值310亿元，增长9.5%；固定资产投资968.6亿元，增长16.5%，增速稳居全区前列；外贸进出口总额1410亿元，增长15.8%，进出口总额、出口总额继续位居全区第一，边境小额贸易进出口总额占全区比重近八成、全国比重近三成，对外贸易持续领先全区；社会消费品零售总额145.8亿元，增长11%；居民消费价格指数上涨1.6%，控制在目标范围内；城镇登记失业率2.14%，比自治区目标控制线低2.06个百分点；城乡居民人均可支配收入增长10.6%。总体看，全市经济运行总体平稳、稳中有进、稳中向好。

（二）扩大开发开放，口岸优势巩固提升。开放合作平台加快建设，凭祥重点开发开放试验区总体规划、若干政策等顺利实施，试验区建设进入快车道。凭祥综合保税区扩容提量，入区企业进出口总额340亿元，增长80%。边境加工业成为投资新热点，完成产值53.68亿元，增长66.3%。先行先试成效突出，友谊关口岸公共服务平台子系统上线运行；中越跨境劳务合作试点工作启动实施，设立3个境外边民务工管理服务中心。互联互通不断加强，友谊关公路口岸获批扩大开放，爱店口岸升格基础设施通过自治区验收，大新硕龙口岸升格为国家一类口岸，实现边境县（市）一类口岸全覆盖；水口至驮隆中越界河二桥等跨境通道项目开工建设，中越友谊关—友谊国际口岸货运专用通道通车运行。

（三）推进绿色发展，文化旅游提质增速。依托良好绿色生态优势，加快发展全域旅游，促进文化旅游提质增速。持续加强生态建设，主要江河地表水和集中式饮用水水质达标率均达100%，空气质量综合排名全区第三，龙峡山成功晋升为国家级森林公园，大新黑水河湿地景区被评为“中国最具原生态景区”。景区景点提档升级，中越德天—板约瀑布跨境旅游合作区加快建设，左江花山音画夜游将于春节前实现运营，德天跨国瀑布即将成功创建国家5A级旅游景区，新增7家国家4A级旅游景区、3家四星级旅游饭店、3家广西生态旅游示范区、13家四星级乡村旅游区（农家乐）。旅游新业态加快发展，成功召开首届中国崇左民宿大会，西坡、大乐之野、过云山居等一批知名民宿企业重点项目签约落地。文化旅游融资渠道进一步拓宽，与云锋基金、东方文旅分别共同设立100亿元、50亿元旅游产业基金，引入中国旅游集团参与德天跨国瀑布景区和花山岩画景区建设运营。全年接待游客总人数和旅游总消费分别增长23.2%、25.8%。

（四）加快转型升级，产业体系日趋多元。深入推进供给侧结构性改革，加快产业转型升级，三次产业结构调整为20.6：43：36.4。糖锰“二次创业”深入实施，崇左东亚糖业循环经济综合利用项目开工建设，中信大锰镍钴锰酸锂三元正极材料项目一期竣工投产，制糖业、锰业、木材加工业规模以上产值分别增长16.9%、13.5%、115.2%。新兴产业加快发展，龙州生态氧化铝、中泰产业园稀土金属等项目开工建设，南国铜业等项目稳步推进，稀土下游产业产值实现零的突破，越香园酸奶、金亚新能源电动车一期等项目竣工投产。

新增49家规模以上工业企业，创历史新高。现代农业创新发展，粮食总产量51.16万吨，建设“双高”基地面积41.2万亩，新增25个现代特色农业示范区，凭祥美人椒种植示范区列入第九批国家农业标准化示范项目。现代服务业快速发展，龙州县、扶绥县获评为全国电子商务进农村综合示范县，凭祥综合保税区、凭祥红木交易市场列入全区首批现代服务业集聚区名单。园区建设稳步推进，海峡两岸产业合作区崇左产业园、广西·中国糖业产业园启动建设，中泰产业园与泰国六个园区结成“两国多园”联袂发展格局，龙赞东盟国际林业循环经济产业园荣获“中国十大木业园区”称号。全市园区工业总产值702亿元，增长36%，占全市工业总产值76.1%。

（五）狠抓项目建设，发展基础日益夯实。完善重大项目推进机制，以“双10”项目为抓手，落实重大项目前期工作经费2.4亿元，加快推进交通、城建、水利水运等一批项目建设，左江治旱驮英水库及灌区工程、崇左至水口高速公路、宁明至凭祥产业大道等项目加快建设，南宁至崇左城际铁路、隆安至硕龙高速公路、大新德天至宁明花山公路、扶绥山圩至中泰产业园一级公路等项目开工建设，全年新开工项目2250项、续建624项、竣工投产1608项。重大项目拉动作用明显，总投资5000万元以上重大项目244个，同比增加95个，完成投资280亿元，增长79%，拉动投资增长15个百分点。园区基础设施不断完善，建成标准厂房75.5万平方米。融资规模稳步扩大，完成重大项目融资439.38亿元，增长13.4%。民间投资趋势良好，完成投资410亿元，增长15%。招商引资成效显著，实施精准招商、联盟招商，开展“桂商崇左行”“台商崇左行”“东方文旅联盟首届年度峰会暨广西崇左旅游投资发展大会”等系列招商活动，全年区外招商引资项目到位资金增长10.1%，其中全口径实际利用外资增长47.1%。

（六）坚持精准施策，脱贫攻坚纵深推进。多措并举打好脱贫攻坚战，严格落实“4321”干部精准帮扶工作机制，创新开展精准帮扶网格化管理，扎实推进扶贫基础性工作。落实财政资金17.93亿元投入扶贫开发，预计全年实现9.5万贫困人口脱贫、83个贫困村出列、1个县脱贫摘帽，全面完成自治区下达的脱贫攻坚任务。产业扶贫“造血”功能不断增强，种植业覆盖带动6万多户贫困户实现增收，牛羊养殖业覆盖带动2.25万户贫困户实现年人均增收700元，“扶贫车间”模式成为全区脱贫攻坚工作新亮点。贫困村集体经济加快发展，设立村集体经济发展基金，83个预脱贫村集体经济年收入均达2万元以上。贫困群众住房得到有效保障，易地扶贫搬迁项目竣工住房7837套，搬迁入住1.81万人；危房改造项目开工8417户，竣工7915户。“百企联百村·万人帮万户”活动有效开展，118家非公企业参与帮扶235个贫困村。扶贫惠农政策有效落实，累计为3.19万户贫困户发放扶贫小额贷款14.37亿元，对贫困户家庭学生实施15年免费教育，投入4983万元落实贫困患者住院费用兜底保障制度，贫困群众基本医疗保险参保率100%。与广东省江门市建立扶贫协作关系，获得第一批帮扶资金8000万元。全力支持龙州县脱贫摘帽，有望通过国家核验，打响广西国家扶贫开发重点县脱贫摘帽第一炮。

（七）统筹城乡发展，新型城镇化稳步推进。围绕建设山水园林城市目标，加快新型城镇化建设，促进城乡协调发展。中心城区建成区面积扩大2.38平方公里，城镇化率提高到38.65%。中心城区提升工程加快实施，重点推进7大系列39个重大城建项目建设，环城东路改扩建二期工程、骆越公园等项目建成使用，崇左大桥、市体育中心等项目加快建设，崇善大道、中心城区水系连通工程等21个项目开工建设。城市宜居度不断提升，城市人均公园绿地面积13.7平方米，位居全区第二。城乡一体化统筹推进，江州区新和镇列入全国特色小镇示范建设范围，51个乡土特色示范村屯加快建设，3个单位、1镇1村荣获“全国文明单位（村镇）”。“宜居乡村”建设深入开展，创建8个市级美丽乡村综合示范村，全市城镇生活垃圾无害化处理率91%，城镇污水集中处理率90%，“美丽崇左”建设再上新台阶。

（八）加快改革创新，发展活力不断增强。深化行政审批制度改革，审批环节压缩35.1%、期限压缩53.2%；实施“多证合一、一照一码”商事制度改革，实现“49证合一”，一般件办理平均用时从50个工作日压缩至3个工作日。沿边金融改革创新发展，新建8个互市贸易结算服务中心，在全区率先实施越南盾现钞跨境调运业务，跨境人民币结算量稳居全区前列；实施跨境劳务人员人身意外保险，签发全国首单机动车出境综合商业保险。农村改革深入推进，农村土地承包经营权确权可发证率95.7%。中泰产业园、凭祥边境经济合作区等园区体制机制改革稳步推进。加快制糖企业战略重组，推动东亚糖业成功收购宁明丰浩糖厂。深入实施创新驱动发展战略，成立崇左高新技术产业开发区和广西—东盟商标品牌创新创业基地，建成崇左首个自治区级科技企业孵化器，新增6家高新技术企业，每万人口发明专利拥有量增长37%，新增14个广西名牌产品。

（九）加强民生建设，群众福祉持续增进。全面完成十项为民办实事工程。全市民生支出182.69亿元，占一般公共预算支出比重为82.4%。教育事业加快发展，广西城市职业学院崇左校区、北大崇左附属实验学校、麦水桥中学开学招生，广西东盟国际职业技术学院、广西大学附属中学崇左校区开工建设，大新县、宁明县、天等县通过国家义务教育均衡发展督导评估。文化惠民工程扎实推进，62个村级公共服务中心竣工验收，“互联网+”有线电视进村入户工作走在全国前列。健康崇左建设加快推进，市县乡三级公立医院综合改革实现全覆盖，建立5个三二医联体、28个县域医疗共同体，蝉联“广西卫生城市”称号。社会保障水平稳步提高，棚户区改造开工5972套、基本建成7233套、分配入住3687套，全市城乡居民基本养老保险、医疗保险参保率分别达98%、97%，各项社会保险待遇发放率100%，城乡低保标准和救助水平持续提高，人人享有社保愿景基本实现。就业和物价保持稳定，安全生产形势持续向好，食品药品监管深入开展，公共安全防控扎实有效，获评2014—2016年度“广西无传销城市”。平安崇左建设不断加强，社会治安综合治理有效开展，双拥共建扎实推进，全市群众安全感、社会稳定动态八项工作综合总分继续位居全区第一，获评“2015—2016年度建设平安广西活动先进市”，凭祥市荣获全国社会治安综合治理最高奖“长安杯”。

（十）转作风提效能，政府建设持续加强。加快政府机构改革，成立市园林局，在中泰产业园设置行政审批局。编制和优化行政权力运行流程，完成市本级电子政务外网横向网络建设，行政效能进一步提高。认真抓好中央第三巡视组对广西“回头看”反馈意见整改工作。严格落实中央八项规定实施细则，市本级“三公”经费同比下降4.6%。依法接受人大及其常委会监督，自觉接受人民政协民主监督，人大代表建议和政协提案办结率100%。认真落实全面从严治党要求，推动“两学一做”学习教育常态化制度化，持之以恒正风肃纪，法治政府建设稳步推进，廉政建设扎实开展，政府公信力和执行力不断提高。

一年来，我市人力资源、审计、法制、司法、计划生育、统计调查、国有资产监管、新闻出版、体育、外事侨务、二轻、供销、民族宗教、涉台事务、机关事务管理、接待、信访、调处、驻邕管理、驻京联络、地震、人防、边防、税务、银监、供电、无线电管理、农机、气象、水文、消防、海关、海事、检验检疫、档案、保密、国家安全、老龄、地方志等工作取得新进展。银行、保险、邮政、通信、石油、烟草、盐业等行业和工会、共青团、妇联、工商联、科协、文联、侨联、残联、社科联、红十字会、法学会、贸促会等群团组织主动作为、扎实工作、不断进步，为全市经济社会发展作出了积极贡献。

各位代表，过去一年所取得的成绩来之不易。这是市委统揽全局的结果，是市人大、市政协监督支持的结果，是全市广大干部群众奋力拼搏的结果，是离退休干部亲切关怀的结果，是驻崇左人民解放军、武警部队和社会各界鼎力支持的结果。在此，我代表市人民政府，向全市各族人民，向人大代表、政协委员，向各民主党派、工商联、无党派人士、人民团体，向驻崇左人民解放军、武警部队官兵，向所有关心支持崇左发展的社会各界，表示衷心的感谢和崇高的敬意！

在肯定成绩的同时，我们也清醒看到，我市发展中仍存在不少困难和问题。经济企稳向好基础还不够牢固，一些发展不平衡不充分的问题较为突出；发展质量效益还不高，传统产业转型升级步伐较慢，新动力还接续不上；生态环境保护压力不断加大，脱贫攻坚进入啃“硬骨头”关键时期，民生领域还有不少短板；政府自身建设仍需加强，部分干部抓落实的能力有待提升等等。对此，我们必须高度重视，采取有力措施，认真加以解决。

二、 2018年工作安排

今年是贯彻党的十九大精神的开局之年，是我国改革开放40周年，是决胜全面建成小康社会、实施“十三五”规划承上启下的关键一年，是广西壮族自治区成立60周年，是崇左建市15周年，做好今年工作意义重大。党的十九大作出了中国特色社会主义进入新时代的重大判断，自治区党委十一届三次全会对持续营造“三大生态”、加快实现“两个建成”、扎实推进富民兴桂、奋力谱写新时代广西发展新篇章进行了系统部署，市委四届三次全会提出要坚定不移推进“两篇大文章”“四大攻坚战”战略任务，为崇左加快发展提供了根本遵循。当前，崇左正处在决战脱贫攻坚、决胜全面小康的关键时期。我们要牢牢把握我市作为革命老区、民族地区、边疆地区、贫困地区这个最大市情，牢牢把握后发展欠发达这个最大实际，牢牢把握正处于工业化初期向中期过渡时期和爬坡过坎、补齐短板的艰苦创业期这个阶段性特征，立足市情实际，抢抓发展机遇，以饱满的工作热情、务实的工作作风、崭新的工作姿态，努力推动高质量发展，不断满足人民日益增长的美好生活需要，开创崇左经济社会发展新局面。

根据中央和自治区精神，结合我市实际，今年政府工作的总体要求是：全面贯彻党的十九大精神和中央、全区经济工作会议精神，以习近平新时代中国特色社会主义思想为指导，坚持稳中求进工作总基调，坚持新发展理念，紧扣我国社会主要矛盾变化，按照高质量发展的要求，统筹推进“五位一体”总体布局和协调推进“四个全面”战略布局，坚持以供给侧结构性改革为主线，保持战略定力，坚定不移做好“两篇大文章”、打好“四大攻坚战”，统筹推进稳增长、促改革、调结构、惠民生、防风险各项工作，大力推进改革开放，加强和改善民生，努力探索具有崇左特色的高质量发展之路，促进经济社会持续健康发展，为奋力谱写新时代崇左发展新篇章奠定坚实基础，以优异成绩迎接建市15周年。

根据总体要求，今年经济社会发展的主要预期目标是：地区生产总值增长8%—8.5%；财政收入增长4%；固定资产投资增长13%；规模以上工业增加值增长8%；外贸进出口总额增长10%；社会消费品零售总额增长10%；居民人均可支配收入增长8%；居民消费价格指数涨幅控制在自治区提出目标以内；单位地区生产总值能耗下降2%；城镇登记失业率控制在4.2%以内。

为实现上述目标任务，贯彻高质量发展要求，我们要保持战略定力，持续推进“两篇大文章”“四大攻坚战”战略任务，推动全市经济社会持续健康发展。要重点抓好以下八个方面工作：

（一）加快推动口岸经济优化升级。坚持大胆探索，先行先试，用足用活国家政策，加快“引进来”和“走出去”，推动开放层次加深、质量提升，促进口岸经济大繁荣大发展。

完善提升开放平台建设。全力推进凭祥重点开发开放试验区建设，加快凭祥综合保税区二期等园区建设，加快实施一批标志性项目，推动试验区建设早见成效、早出成果。制定并完善相关政策，把海峡两岸产业合作区崇左产业园打造成为台资企业参与“一带一路”建设的重要平台。加快中越凭祥—同登跨境经济合作区、中国—东盟边境贸易龙州国检试验区、龙州边境经济合作区申报建设，力争凭祥边境经济合作区扩区调整获得国家批复。

推动对外贸易扩量提质。巩固提升边境贸易，优化一般贸易，提高加工贸易占比，促进各种贸易方式协同发展。深入实施“加工贸易倍增计划”，完善支持边境加工业发展的优惠政策，积极发展木材加工、特色食品加工、冰鲜水产品加工等边境加工业，加快盐津铺子二期、凭祥红木创意产业园一期等项目建设，确保金梧桐坚果果干加工基地等项目竣工投产。推动“广贸通”边境贸易综合服务平台全面投入运营，开展凭祥市场采购贸易方式试点工作。加强出口品牌建设，大力培育自营出口企业。加快推动互市贸易改革，深化贸易结算服务。

优化口岸发展环境。加强口岸基础设施建设，推动水口口岸扩大开放、爱店口岸升格通过国家验收、凭祥（铁路）口岸获批为进境水果指定口岸，推进硕龙口岸升格基础设施建设。加强互联互通建设，加快中越水口—驮隆界河二桥、中新互联互通南向通道（陆路）物流线路等项目建设，扩大“单一窗口”等通关模式实施范围，加快“两国一检”通关模式改革，不断提高通关便利化水平。加快推动跨境劳务试点政策落实到位，进一步规范跨境务工人员及用工单位管理。加快推进跨境动物疫病区域化管理工作，争取尽快获得国家相关部委批准开展试点工作。

加大招商引资力度。大力实施“一把手”招商、以商招商、联盟招商、产业链招商等精准招商方式，进一步加大招商引资力度，全年区外招商引资项目到位资金330亿元以上。加强与越南、泰国等东盟国家交流合作，深化与港澳台及发达地区的区域合作。积极兑现优惠政策，推动项目资金落实到位，建立健全招商快速反应机制和高效协调服务机制，确保一批产业项目落地建设。

（二）加快推动生态文化旅游融合发展。充分发挥优势，积极推动生态与旅游、文化、产业相融合，推进文化旅游提档升级。

加大生态建设和环境保护力度。贯彻“绿水青山就是金山银山”理念，坚持绿色发展，保持良好的生态优势，为创建全区山清水秀自然生态示范市提供坚强保障。狠抓生态建设，做好植树造林和森林抚育工作，加强自然保护区、湿地公园等环境监管。实行最严格的生态保护制度，全面停止天然林商业性采伐，推进国家第二批山水林田湖生态保护与修复工程试点工作。抓好环境保护治理，认真解决环境突出问题，持续推进中央环境保护督察反馈问题和崇左市“专业查污”发现问题的整改工作。全面打好污染防治攻坚战，持续实施大气污染防治行动计划，深入落实“水十条”“土十条”，全面实施“河长制”和最严格水资源管理制度考核。强力推进节能减排，全年能源消费增量控制在13.78万吨标准煤以下，单位地区生产总值二氧化碳排放降低1.57%。建立完善生态环境监管体制机制，用制度保护生态环境。

推动旅游提质扩量。深入实施“发现山水崇左·圆梦别样桂林” 文化旅游发展战略，大力发展全域旅游，加快打造一龙头、一中心、若干个旅游集聚区、若干条旅游发展带，力争用3—5年把崇左建设成为国内知名旅游目的地。加快完善中心城区“吃住行游购娱”六大功能和旅游集散中心建设，进一步引商气、聚人气、旺地气。继续与中旅集团、云锋基金、东方文旅、泛海集团等知名企业对接合作，全力推进一批旅游重大项目。年内开工建设花山博物馆、周氏兄弟艺术馆、东方文旅城、东方文旅小镇等项目，启动建设中旅集团德天小镇项目，确保左江花山音画夜游和大型实景演出项目建成运营、花山剧场投入使用。推进中越德天—板约瀑布跨境旅游合作区尽快实现跨境旅游。抓好特色旅游名县和创A创星工作，年内争取凭祥市成功创建国家全域旅游示范区，宁明县、龙州县成功创建为广西特色旅游名县，花山岩画景区通过国家5A景区旅游资源景观质量评定，年内新增国家4A级旅游景区5家、四星级旅游饭店2家、四星级以上乡村旅游区（农家乐）3家。加快发展旅游民宿，推动西坡、大乐之野等民宿项目年内建成营业，打造广西旅游民宿集群。探索白头叶猴生态公园、左江斜塔等景区运营管理改革创新，推行所有权、经营权分离，提高旅游景区服务品质和运营效益。

加强配套建设和营销宣传。进一步完善旅游交通、游客服务（咨询）中心、汽车旅游营地等配套设施。推进“厕所革命”，年内新建改建旅游厕所32座。创新旅游宣传营销模式，加强与旅游行业知名电商合作，加大广告投放力度，形成线上线下多渠道联动营销模式。强化旅游软环境建设，提高旅游服务质量。加强崇左土特产设计包装，推动形成系列旅游商品。探索引入影响力大的国际体育赛事。深化与东盟国家旅游推广合作，拓展国际旅游市场空间。全年接待游客总人数、实现旅游总消费分别突破3000万人次、300亿元大关。

（三）提升实体经济发展水平。实体经济是国民经济的脊梁。要把发展的着力点放在实体经济上，加快推进产业转型升级，加快培育新的经济增长点，不断提升发展的质量和效益。

加快产业转型升级。全力推进糖锰“二次创业”，加快木业转型发展，提升产业价值链和产品附加值。大力发展蔗糖深加工和糖业循环经济，加快广西·中国糖业产业园建设，推动崇左东亚糖业循环经济综合利用、湘桂活性干酵母等项目竣工投产。推动锰业向高端化发展，开工建设中信大锰镍钴锰酸锂三元材料二期等项目，加快建设新振锰业中低碳锰铁合金一期等项目。加快推动龙赞东盟国际林业循环经济产业园、广西山圩产业园、凭祥边境经济合作区林产业加工区等园区建设，年内竣工投产15个以上木材加工项目。大力发展新兴产业，加快龙州生态氧化铝、中铝国盛稀土二期、中泰产业园稀土金属等项目建设，确保南国铜业一期、琦泉生物质发电、金象新能源一期、广西劲高电子科技等项目竣工投产。大力发展现代服务业，加快推进自治区服务业重大项目库、行业龙头企业名录库、现代服务业集聚区“两库一区”建设。大力发展商贸物流业，推进凭祥市跨境冷链物流、花山空港小镇等项目建设，开业运营爱琴海购物公园，积极申报两岸冷链物流产业合作试点城市，促进快递业健康发展。大力发展旅游、健康、养老等生活性服务业，推进养老服务业综合改革试验区建设，探索医养结合新路子。加快发展电子商务，进一步完善市、县、乡、村物流体系和电商服务体系，推动凭祥市纳入全国电子商务进农村综合示范县创建范围，推进中国—东盟（凭祥）电商产业园等跨境电商项目建设。

深入实施创新驱动发展战略。加快推进崇左高新技术产业开发区建设，加快建设科技企业孵化器、众创空间等创新平台，年内建成自治区级高新区。深化与中南大学、广西大学等高校产学研合作，发挥院士专家服务中心作用，加强技术攻关和科技成果转化，在糖业、锰业、木业、新材料等重点领域的关键技术上取得新突破。深入实施质量强市战略，大力推动品牌和标准化建设。

增强金融服务实体经济的能力。推动政银企常态化合作，引导金融机构扩大对重点产业、重点项目信贷投放规模。大力发展普惠金融、绿色金融、科技金融，全面提升政府性融资担保业务能力和水平，着力缓解中小微企业融资难题。加大“引金入崇”力度，年内引进更多金融机构入驻崇左。

优化营商环境。进一步增强服务企业意识，以打造稳定、公平、透明的营商环境为目标，按照市场化配置要求，营造有利于企业发展的法治环境、政策环境、市场环境。严格执行市四家班子领导联系服务企业制度，建设崇左市服务企业信息平台，开展“市长服务企业日”活动，定期召开企业建设、生产、发展协调会，解决企业的困难和问题，扶持企业不断发展壮大。落实非公经济发展政策，引导非公企业增强自主创新能力，加快企业发展。

（四）全力打好脱贫攻坚战。动员全市全社会力量，坚持精准扶贫、精准脱贫，全力攻克深度贫困地区脱贫攻坚任务，全面完成自治区下达的年度脱贫摘帽任务。

抓实脱贫攻坚基础性工作。进一步规范第一书记、驻村工作队、村“两委”、帮扶单位、帮扶联系人管理。坚持精准帮扶网格化管理，重点推进以村为单位的驻村专业扶贫工作队建设，不断提升“一户一册一卡”填写、扶贫项目落实和群众满意度工作水平。认真抓好精准脱贫退出户跟踪观察和扶持工作，加强扶贫信息动态管理和扶贫档案规范管理。强化领导干部扶贫政策学习，扎实推动扶贫政策落实到位，紧紧围绕“两不愁三保障”目标，列出教育、医疗、住房等扶贫政策清单，并逐项落实到户到人。

着力攻克脱贫攻坚的重点难点。强化市领导包县、县级领导包易地扶贫搬迁安置点建设工作责任制，加大推进力度，确保年内实现搬迁25428人。大力发展村集体经济，发挥贫困村集体经济发展基金引导拉动作用，通过发展产业、村企联营等措施，确保所有预出列和已出列村集体经济年收入达3万元以上。加强贫困地区基础设施建设，实施村屯道路、危房改造等项目，补齐发展短板。

加快发展扶贫产业。大力发展“种养贸游工”五大扶贫产业，确保贫困群众有稳定收入来源。在巩固发展甘蔗种植基础上，引导贫困户发展特色水果、秋冬蔬菜等特色效益农业。在抓好牛羊养殖带动脱贫的同时，稳定生猪养殖，大力发展林下养殖、稻田综合种养等“短、平、快”项目，扩大养殖扶贫成效。扶持发展边民互助合作社，引导贫困边民参与边境贸易、边贸加工。支持贫困群众通过入股、务工、开发特色农产品和手工艺品等方式参与乡村旅游。大力推广“扶贫车间”模式，加快打造养殖、工业加工、休闲旅游和贸易“扶贫车间”，带动更多贫困群众就近就业、脱贫致富。同时，坚持专项扶贫、行业扶贫和社会扶贫联动，形成全社会共同参与扶贫的强大合力。加强与广东省江门市扶贫协作，用好相关投资优惠政策，加快推进双方协定项目建设，确保粤桂扶贫协作工作取得实效。进一步深化“百企联百村·万人帮万户”活动，提高民营企业参与扶贫的成效。

（五）全面实施乡村振兴战略。按照产业兴旺、生态宜居、乡风文明、治理有效、生活富裕的总要求，大力实施乡村振兴战略，推进城乡协调发展。

加快推进农业现代化。严格落实耕地保护制度，加强粮食生产功能区建设。坚定不移抓好甘蔗生产，全面落实400万亩糖料蔗生产保护区划定任务，完成64万亩“双高”基地建设任务。深入推进农业供给侧结构性改革，狠抓农产品标准化生产、品牌创建、质量安全监管，培育发展有机循环农业等新兴产业。积极推广标准化养殖，新增自治区级标准化牛羊养殖示范场5家。强力推进一批现代农业示范区建设，新增区县乡三级示范区（园）99个、村级示范点250个，把示范区建成实施乡村振兴战略的样板区。加快培育新型农业经营主体，年内新增农业企业30家、农民合作社250家、家庭农场70家。加快发展农业机械化，耕种收综合机械化率达62%。加强农产品初加工、精深加工及综合利用，支持发展“互联网+现代农业”，推动农村一二三产业融合发展。

深化农村综合改革。完善承包地“三权分置”制度，加快土地流转及“小块并大块”，推进农村集体产权制度改革，全面完成农村土地承包经营权确权登记。加快农村金融改革，支持发展村镇银行、小额贷款公司等农村金融服务组织，支持金融机构开办创业贷款、农户联保贷款等业务。加快推进农村信用体系建设，持续开展农村信用“四级联创”工作，年内新增“三农”金融服务室60个以上。支持发展特色农业保险，积极开展肉牛、母牛养殖保险，加快实施糖料蔗价格指数保险。加大农村实用人才带头人、家庭农场主、农民合作组织等新型农业经营主体培训力度，培育造就一支懂农业、爱农村、爱农民的“三农”工作队伍。加快推进“美丽崇左”乡村建设升级版，深入实施百村示范百村整治“双百工程”，建设10个星级村，启动“幸福乡村”建设。

大力发展县域经济。按照县域经济“三区一镇”发展模式，积极引导各县（市、区）按照主体功能区规划，加快构建特色鲜明的县域特色产业体系，争创1个先进县、1个进步县。大力推进县域工业园区建设，培育优势骨干企业和品牌产品。坚持“一主二副两带”规划，加快建设扶绥、凭祥两个市级副中心城市，抓好凭祥国家中小城市综合改革试点和大新新型城镇化示范县建设，加快凭祥—宁明—龙州一体化发展。大力实施中心镇、特色镇示范工程，抓住自治区大力支持特色小镇建设的契机，加快建设新和甜蜜小镇、水口边贸小镇、山圩木艺小镇，积极打造硕龙跨国旅游小镇。推进县域重点领域改革创新，在土地利用、行政审批等方面赋予县级更多自主权，激发县域经济发展活力。

（六）强化基础设施重大项目建设。以重大项目为重要抓手，加快城建、交通、水利、能源等领域的一批重大项目建设，提高基础支撑能力，增强发展后劲。

积极扩大有效投资。全力推进市层面以上统筹推进的251项重大项目建设，年内完成投资301.5亿元以上。全市安排重大项目前期工作经费2.4亿元以上，力争不断有项目新开工。加大项目储备力度，推动更多项目纳入自治区重大项目盘子。加强园区水、电、路、标准厂房等基础设施建设，确保年内各重点产业园核心区达到“五通一平”。重点推动中泰产业园新材料产业园污水处理厂及配套管网等项目建成运行。

加快提升中心城区品质。加快推进花山风景名胜区规划报批工作和城市总体规划重新编制，年内编制完成中心城区和各县城区（凭祥市）近期建设区域控规。大力实施中心城区提升工程，加快完善中心城区经济中心、商贸中心、文化教育中心、旅游集散中心“四个中心”城市功能，中心城区建成区面积增加2平方公里以上。加快中泰产业园、城西片区开发建设，推进产城融合，提升中心城区经济实力。加大老城区改造力度，加快建设太平古城改造等项目，推动沿山路西段改造等路网项目建成通车。继续抓好7大系列项目建设，开工建设华润商业综合体、金龙湖公园等项目，确保崇左大桥、市体育中心、崇善大道等项目建成使用。加快创建国家园林城市和海绵城市，推进龙峡山森林公园、龙腾湖东园、金鸡山森林公园等城市公园建设，结合棚户区改造建设一批城市小绿地、小广场。进一步优化城区供水、供气、供电、污水处理等设施，城镇污水处理率达92%以上。

加快现代交通体系建设。推动南宁至崇左城际铁路崇左段全线动工。积极开展崇左至爱店高速公路前期工作，加快建设崇左至水口高速公路、隆安至硕龙高速公路、大新德天至宁明花山公路、扶绥山圩至中泰产业园一级公路、南友高速公路花山互通工程等项目，确保南宁新江经吴圩至崇左扶绥一级公路崇左段等项目建成通车。加快县乡联网工程、农村公路生命安全防护工程建设，抓好二级路及以下等级公路维修改造提级工作。

加快水运水利基础设施建设。加快推进山秀船闸扩能工程、渠珠水利枢纽工程、左江治旱黑水河灌区等项目前期工作，加快驮英水库枢纽及灌区总干渠建设，开工建设4大干渠，推动中心城区备用水源工程、河湖水系连通一期工程等项目竣工使用、崇左港中心港区濑湍作业区试运行。大力推进中央财政小型农田水利、农村饮水安全巩固提升工程等民生水利建设，新增供水受益人口3.3万人。

抓好能源信息基础设施建设。继续实施县县通天燃气工程，新增1个县通天燃气。加强电网建设，推进2个主网项目、22个配网项目、159个农网改造升级工程建设。加强宽带和通信基站建设，确保全市行政村通宽带及4G信号全覆盖。

（七）加快推进全面深化改革。深入推进供给侧结构性改革和重点领域、关键环节改革，更好发挥改革牵引作用，为经济平稳发展和社会稳定注入新活力。

深化供给侧结构性改革。加快“三去一降一补”任务落实，着力增强改革的系统性、整体性、协同性。加快推进大新雷平永鑫公司、世纪飞龙公司和天等东糖公司战略重组工作，提高糖业产业集聚力和竞争力。加快建立多主体供给、多渠道保障、租购并举的住房制度，努力让全体人民住有所居。全面落实各项减免税政策，引导企业参与全区电力直接交易，加快组建崇左市本级售电公司，探索中泰产业园增量配电业务试点建设，深化崇左市电力体制改革，切实降低企业用电成本。健全政府性债务管理体系，优化政府债务结构。加快完善地方金融监管体制，严厉打击非法集资等违法违规金融活动，妥善处置银行不良贷款，有效防控金融风险。从人民群众迫切需要解决的突出问题着手，补齐公共服务、基础设施和环境保护等方面短板。

深化行政审批制度改革。深入推进简政放权，加大“放管服”力度，压缩审批环节，优化政务大厅审批窗口设置。扎实开展“互联网+政务服务”工作，推动实体政务大厅向网上办事大厅延伸。推进中泰产业园“一站式”审批服务平台建设。深化“证照分离”改革，推进行政审批中介服务事项管理改革。深入推进商事制度改革，对“多证合一”登记改革事项实行动态调整。强化“双随机、一公开”监管，完善以信用监管为核心的事中事后监管体系。推行企业“先落地后缴费”制度，减少企业生产经营成本。加大综合行政执法改革力度，推动综合执法向乡镇延伸。

稳步推进财税金融体制改革。进一步完善预算管理制度，扎实推进全口径政府预算管理，加强地方政府债务预算管理和限额管理。稳步推进财政事权、支出责任划分和各项税收制度改革，加大力度培育地方主体税种。大力推广政府和社会资本合作（PPP）等模式，设立产业发展基金，积极推动多元化投资。深化沿边金融综合改革，积极创建“一带一路”广西门户保险创新综合试验区，推动筹建边贸银行，拓展中越跨境金融合作，探索建立人民币与越南盾现钞调配与清算中心，推动互联网金融支持外贸发展，提升跨境人民币结算业务水平。

统筹推进重点领域改革。继续推进凭祥综合保税区、中泰产业园、凭祥边境经济合作区等园区体制改革，提升园区发展活力。深化国企国资改革，加快组建崇左市五大国有集团公司。统筹推进文化、科技、统计、供销、司法、监察、盐业监管等体制机制改革，健全改革落实机制，确保改革终端见效。

（八）着力提高保障和改善民生水平。抓住人民最关心最直接最现实的利益问题，扎扎实实推进解决，不断满足人民群众日益增长的美好生活需要，提升群众获得感、幸福感、安全感。

优先发展教育事业。深入开展教育基础建设，投入资金14亿元以上新建、迁建和改扩建一批学校。大力提升中心城区教育质量，开工建设崇左高中扩建、北大崇左附属实验学校、江州高中等项目，推进广西东盟国际职业技术学院、广西大学附属中学崇左校区建成使用。深化教育领域综合改革，继续实施基础教育质量提升工程，推进普及高中阶段教育攻坚计划和学前教育三年行动计划，推动扶绥县和江州区义务教育均衡发展通过国家评估认定，积极发展民办教育，确保学前三年毛入园率、义务教育巩固率、高中阶段毛入学率均达到自治区目标值。深化职业教育改革，推进南宁地区教育学院转制和新校区建设，支持广西城市职业学院、广西理工职业技术学院升本工作，支持广西民族师范学院转型发展。加强老年大学建设，提升老年大学办学水平。

加强就业和社会保障工作。实施积极的就业创业政策，鼓励创业带动就业，年内城镇新增就业1.4万人以上，新增农村劳动力转移就业3.1万人以上，高校毕业生就业率90%以上。全面加强社会保障，深化机关事业单位养老保险制度改革，完善职工基本医疗保险制度，巩固城乡居民大病保险制度；加强社会救助体系建设，切实保障困难群众基本生活，做到应保尽保；扎实推进保障性住房建设，开工建设棚户区（危旧房）改造6472套。

大力发展文化体育事业。继续开展“三月三”花山文化节、花山岩画国际论坛等系列活动，举办第二届文学艺术“花山奖”评审活动。稳步推进村级公共服务中心建设。加强“扫黄打非”工作。全面推进软件正版化工作。推进全民健身与全民健康深度融合，推动企事业单位、学校公共体育场馆免费或低收费向社会开放。精心组织承办好广西第十四届少数民族传统体育运动会。

加快健康崇左建设。加快国家卫生城市创建工作。深化医药卫生体制改革，探索建立现代医院管理体制。完善分级诊疗制度，推动广西民族医院与天等县、大新县人民医院，崇左市人民医院与龙州县人民医院组建紧密型医联体，乡镇卫生院参与县域医共体建设达50%以上。全面推行公立医疗机构药品采购“两票制”。加快推进市人民医院创建“三甲”工作。加快市人民医院内科综合楼、市中医壮医医院整体搬迁等项目建设。全面优化计划生育服务管理，创建“全国幸福家庭示范市”。

加强和创新社会治理。加强社会治安综合治理，开展禁毒严打整治专项行动，依法打击和惩治各类违法犯罪活动。推进信访工作制度改革，健全依法维权和多元化解矛盾纠纷机制。进一步完善综治中心建设，加快建设“雪亮工程”综治视联网，升级改造“天网”工程，立项建设崇左市政法大数据中心。加强边境管控，加快建设边境拦阻设施。强化食品药品安全监管，创建“广西食品安全示范城市”。全面加强安全生产，健全综合应急救援体系，提高突发公共事件应对处置能力。

继续实施为民办实事工程。筹措资金45亿元以上，实施社保惠民、健康惠民、教育惠民、水利惠民、安居惠民、农补惠民、生态惠民、文化惠民、扶贫惠民、科技惠民等十大类为民办实事工程。

深入实施“人才强市”战略。深入开展“南疆国门聚英才”行动，加快打造“名校学子边关行”引才品牌，加快引进一批高层次、高技能和紧缺急需人才。建设“崇左市振兴左江革命老区青年智库研究会”育才平台，培育一批知识型、技能型、创新型劳动者。

加强民族宗教工作。认真贯彻落实民族宗教政策，深入开展民族团结进步创建活动，继续大力实施兴边富民行动。做好民族语文工作。依法管理宗教事务，维护宗教活动正常秩序和宗教界和谐稳定。

支持国防和军队建设。深化全民国防教育，加强国防动员、国防后备力量和人民防空建设，巩固全国双拥模范城创建成果，提升双拥共建质量，推进军民融合深度发展。

三、 全面加强政府自身建设

深入贯彻落实党的十九大精神，坚持以习近平新时代中国特色社会主义思想为指导，坚定维护核心，牢固树立“四个意识”，以从严治党统领政府各项工作，努力打造人民满意的政府。

（一）坚持依法行政，建设法治政府。坚持依宪施政、依法行政，规范政府行为，严格规范公正文明执法，切实维护人民权益和社会秩序。加强普法工作，全面提升法治化水平。自觉接受人大及其常委会监督和政协民主监督，切实做好人大代表建议和政协提案办理工作，让党和人民赋予的权力始终在法治的约束下规范运行。

（二）坚持实事求是，建设诚信政府。坚持做到言必信、行必果，对企业诚信，对人民诚信。开展公务员诚信、守法和道德教育，强化公务员队伍诚信履职意识。加强政府采购、招标投标等重点领域政务诚信建设，提高工作透明度。规范政府招商引资行为，认真履行依法作出的政策承诺和签订的各类合同。大力推进政务公开、信息公开，保障人民群众知情权、参与权、表达权、监督权。

（三）坚持实干担当，建设高效政府。坚持干字当头，坚决落实市委的决策部署，严格执行市委的决定，压实工作责任，强化督查落实，把90%以上的时间和精力放在抓落实上，做到干一件成一件，成一件见实效一件，提升政府执行力和办事效率。坚决整治慵懒散，切实解决不作为、慢作为、乱作为问题，确保各项工作高标准推进、高效率完成。持续加强学习，增强工作本领，不断提高专业化工作水平和破解难题的能力。

（四）坚持严以用权，建设廉洁政府。始终把纪律和规矩挺在前面，严格遵守政治纪律和政治规矩，严格贯彻落实中央八项规定精神，驰而不息整治“四风”。强化廉政建设，进一步加强审计监督，抓好重点领域和关键岗位廉政风险防控，全力打造干部清正、政府清廉、政治清明的政务环境，树立政府清正廉洁的良好形象。

（五）坚持执政为民，建设服务型政府。时刻牢记以人民为中心的发展理念，始终把人民对美好生活的需要作为奋斗目标，认真履行社会管理和公共服务职能，深入推进“放管服”，把更多公共资源向民生倾斜，更好地为基层、为企业、为群众服务，把难事办好、好事办实。

（六）坚持解放思想，建设创新型政府。坚持构建有利于激励创新的机制体制，持续推进政府公共部门改革和完善，营造保护创新的环境。始终保持思想的敏锐性和开放度，积极破除“旧思维”、突破“老框框”，用新理念、新思路、新办法来破解难题、推动发展。

各位代表，新时代开启新征程，新作为书写新篇章。让我们紧密团结在以习近平同志为核心的党中央周围，以习近平新时代中国特色社会主义思想为指导，在市委坚强领导下，以永不懈怠的精神状态和一以贯之的奋斗姿态，开拓进取、真抓实干，不断开创经济社会发展新局面，为做好“两篇大文章”、打好“四大攻坚战”，与全国全区同步全面建成小康社会、谱写新时代崇左发展新篇章作出新的更大贡献！
